# Recognizing and reporting domestic violence: attitudes, experiences and behavior of Dutch dentists

**DOI:** 10.1186/s12903-015-0141-4

**Published:** 2015-12-15

**Authors:** Brigitte A. F. M. van Dam, Wil J. M. van der Sanden, Josef J. M. Bruers

**Affiliations:** Department of Research & Information, Royal Dutch Dental Association (KNMT), Nieuwegein, The Netherlands; Department of Quality and Safety of Oral Health Care, Radboud University Medical Centre, College of Oral Science, Nijmegen, The Netherlands; Academic Centre for Dentistry Amsterdam (ACTA), University of Amsterdam and VU University, Amsterdam, The Netherlands

**Keywords:** GDP, Domestic violence, Mandatory reporting code, Attitudes, Behaviors, Knowledge, Education

## Abstract

**Background:**

On July 1^st^ 2013 the Mandatory Reporting Code Act came into force in the Netherlands, making it compulsory for health professionals to adhere to a reporting code when they suspect patients to be victims of domestic violence (DV) or child abuse (CA). The Royal Dutch Dental Association (KNMT) developed a reporting code for dental professionals (RCD). Moreover, an e-learning module about DV has been developed. A web-survey was conducted to investigate how general dental practitioners (GDPs) deal with the RCD and what their experiences are with (signs of) DV and CA.

**Methods:**

In April 2014 1038 GDPs were invited by e-mail to participate in a web-survey consisting of 24 items, through the KNMT Data Stations Project. The data was analyzed using SPSS (RELIABILITY, CHISQ and ANOVA).

**Results:**

Of all GDPs invited to participate 264 (25 %) responded. 82 % of these GDPs are aware of their obligation to use the reporting code. 54 % of the GDPs are in favor of this obligation. 76 % of the GDPs have taken notice of the KNMT’s RCD and 51 % of the GDPs have implemented the reporting code in one form or another in their practice. 24 % of the GDPs stated having suspected DV during the last twelve months in the case of 2.4 patients on average. 81 % took note of this in the patient’s record and 58 % also took action in different ways. 54 % wants to complete the e-learning module.

**Conclusions:**

Most GDPs are aware of the new legislation and have taken cognizance of the RCD. Even though the majority of GDPs are not opposed to using a reporting code, over half of them have not yet implemented the code in practice. An important factor in this regard seems to be that a substantial minority of the GDPs says they are not sufficiently informed about aspects of reporting a case and about the steps they have to take.

## Background

Domestic violence (DV) can be defined as threatening behavior, violence or abuse between adults who are relatives, partners or ex-partners. It also includes violence or child abuse (CA) from adults to children or abuse from (adult) children to parents [1]. It can take many forms including physical, sexual, emotional, economic or psychological abuse, ranging from subtle to violent, resulting in disfigurement or death, as well as any behaviors that intimidate, threaten, hurt, injure, or wound someone [2].

DV is a serious public health problem, which particularly affects women. Overall, 30 to 35 % of women worldwide have experienced either physical and/or sexual abuse, most of which is intimate partner violence [3, 4]. In the Netherlands each year at least 200,000 people become a victim of serious and/or repeated DV, and 83 % of the offenders is male [5]. Children under 18 years are at risk of CA, including emotional or psychological abuse or neglect [6]. This might occur at home, but also at different kinds of organizations, schools or communities they interact with. International studies show that approximately 20 % of women and 5 to 10 % of men report being sexually abused and 23 % having been physically abused as a child [6]. In the Netherlands in 2010 the prevalence of children being victim of CA was estimated over 119,000 (i.e., 34 per 1000) [7].

In general, the majority of physical injuries from domestic and other forms of violence are inflicted to the head, face or neck [8, 9]. Because there is evidence that many victims interact with or visit oral health care providers, these professionals are able to recognize such abuse [10]. Moreover, family environments with high levels of verbal and physical conflict between members may be implicated in compromised oral health [11]. All the more reason for oral health care professionals to be confident in the identification of DV and uphold their legal and ethical responsibility to record and report it [12–16].

How often primary care clinicians and in particular general dental practitioners (GDPs) suspect DV or CA cannot be concluded from available literature. Ramsay et al. (2012) state that 71 % of primary care clinicians in the United Kingdom have diagnosed one or more new cases of DV in the last 6 months [17]. In 2010, 37 % of Scottish GDPs claimed to have seen at least one suspicious case in their career [10].

However, it appears that in many cases GDPs who suspect DV or CA do not report their suspicions [10, 14, 17–20]. The reasons for this appear to be lack of certainty about the diagnosis, fear of litigation, fear of family violence towards the child, fear of violence directed against the GDP and concern about negative impact on the practice [10, 14, 19]. Lack of knowledge and the feeling of being inadequately informed about issues regarding abuse and protection also play an important role in this process [10, 19, 20]. Survey data in the United States show that although 87 % of GDPs believed that recognizing CA is important, 63 % stated they did not know how to act in such situations, 44 % were unaware of the proper child protection authorities to contact and 95 % reported that they did not receive sufficient education concerning CA in their undergraduate studies [21]. In fact, it appears that dental professionals who have received this kind of education are more likely to screen for DV and to take action when they suspect DV [18, 22, 23].

Meanwhile, in the United States reporting child abuse and neglect is mandatory for health professionals in all 50 states [24]. In several European countries, such as Germany and Sweden, professionals are also required to report suspicion of DV [25]. In the Netherlands, the Mandatory Reporting Code DV and CA came into force on July 1^st^ 2013. This act makes it compulsory for organizations and independent (health) professionals to have a reporting code that meets the statutory requirements [26]. Moreover, they have to promote the awareness and use of the code within their organization. The code describes in five steps what professionals have to do when they suspect DV or CA and how, given their duty of confidentiality, they can reach a sound decision on whether to file a report. The five steps are: 1 Identifying the signs, 2 Peer consultation and, if necessary, consultation with an Advice and Reporting Centre for DV or CA (as of January 2015 combined into ‘Save Home’) or an injury specialist, 3 Interview with the client, 4 Assessing violence and child abuse, 5 Reaching a decision: arranging assistance or reporting a case. The law sets a number of requirements for the content of the reporting code drawn up by an organization. The most important of these is that the code must contain at least the five steps mentioned before.

In order to support oral health care professionals to fulfill their obligations according to the Act, the Royal Dutch Dental Association (KNMT) developed the reporting code for dental professionals (RCD). The RCD contains an information brochure which explains the different forms of DV and neglect, the new legislation, the judicial aspects, how to ask for advice and how to report abuse. The RCD also contains the five step action plan. In 2012 this RCD was sent to all dental practices in the Netherlands. It is up to the GDP to decide how to fulfill the requirements, how to tailor instructions and how to decide what kind of training the dental team needs. In cooperation with two other dental professional associations and with Augeo Academy, the KNMT developed an e-learning module about DV and the reporting code called ‘The Next Page’ [27]. The module became available in 2013. However, in August 2013 the Dutch Health Care Inspectorate (IGZ) concluded that efforts to implement the obligatory reporting code in dental care had been insufficient [28].

The aim of this study is to investigate to which extent Dutch GDPs are aware of the RCD, if they have implemented the RCD and if so, how they implemented it. This study will also investigate how often dentists have had suspicions of DV or CA and how they dealt with their suspicions in the most recent case.

## Methods

### Data collection

By means of its Data Stations Project the KNMT periodically collects data on delivery of oral health care, on practice management and on GDPs’ opinions and views regarding current issues in dentistry in the Netherlands [29]. For this survey in April 2014 a representative group of 466 GDPs who participate periodically in the Data Stations Project were selected. In order to reach sufficient response, this group was enlarged with a random selected group of 572 GDPs. Thus a representative sample of 1038 of the approximately 8600 GDPs in the Netherlands of 64 years of age or younger, received a request by e-mail to complete an online questionnaire. The study was approved by an independent review board of the KNMT. GDPs have been explained that answering and sending in the questionnaire (on a voluntary basis) means that they consent to participate in the study.

The online questionnaire covered 24 items. GPDs were asked about their acquaintance with the RCD and whether or not they had actually perused this or a different reporting code, how they evaluated the content and the ‘look and feel’ of it and if they had implemented the RCD in their practice. Subsequently, their opinions on the effect of the RCD were gauged using four Likert type items. Furthermore, questions were asked about any suspicions of DV or CA in the last 12 months. If GDPs stated that they have had suspicions, some specific details were asked about their professional behavior concerning the most recent case in which suspicion of DV or CA was raised. GDPs who did not respond after 2 weeks and consecutively after 5 weeks, received a reminder e-mail.

### Response and representativeness

Ultimately, 264 (25 %) of the 1038 GDPs in the sample completed the questionnaire. With regard to some individual characteristics, the total group of respondents proved to be fairly representative for the population of GDPs in the Netherlands (Table 1). Admittedly, differences in the population between GDPs who did and GDPs who did not participate in the study were statistically significant (*p* < 0.05), but all associations proved to be very weak (Cramèr’s V < 0.10 and eta^2^ < 0.05).

**Table 1:** Individual characteristics of respondents and of the population of GDPs in the Netherlands (January 2014)

	Respondents	Population^a^
Gender		
Male	67 %	64 %
Female	33 %	36 %
Age*		
29 years of age or younger	5 %	10 %
30–39 years of age	19 %	23 %
40–49 years of age	16 %	19 %
50–59 years of age	43 %	33 %
60–64 years of age	16 %	15 %
Mean age*	*49.0*	*46.4*
University of qualification*		
Amsterdam	35 %	39 %
Groningen	21 %	14 %
Nijmegen	25 %	22 %
Utrecht	15 %	12 %
Foreign country/unknown	4 %	13 %
Year of qualification*		
1979 or before	17 %	13 %
1980–1989	43 %	34 %
1990–1999	15 %	17 %
2000–2009	22 %	27 %
2010–2014	3 %	9 %
Mean year*	*1990.0*	*1993.2*
Geographical location*		
Northern region	11 %	10 %
Eastern region	22 %	18 %
Southern region	22 %	20 %
Western region	45 %	52 %
Membership KNMT*		
Member	91 %	75 %
Non member	9 %	25 %
Total	264	8.653

*Chi-Square: *p* < 0.05, but Cramèr’s V < 0.10

^a^KNMT-dentist administration: registered GDPs in the Netherlands (members and non-members)

### Statistical analysis

The data was analyzed with SPSS. At first by testing if the Likert type items showed internal consistency (reliability) in order to determine if one or more additive scales could be formed.

A Cronbach’s alpha of 0.80 or greater was considered as a criterion for a reliable scale.

Chi-square tests were used to test the correlation between nominal items and by analysis of variance differences between group means were tested.

## Results

### Legal obligation

Most (82 %) respondents stated that they were aware of the fact that since July 1^st^, 2013, dentists and other care workers are compulsory to adhere to a reporting code in case of suspicion of DV or CA. It appears that 54 % is more or less positive about this obligation, while 35 % holds a neutral position. All others (11 %) are negative about the obligation to use a reporting code, most of whom (69 %) stated that they regard themselves not adequately equipped to judge on DV and CA.

### Reporting code for dental professionals (RCD)

The majority (76 %) of GDPs indicated that they received information in some way about the brochure and/or the action plan of the RCD (Table 2). Most GDPs thought the information brochure was clear with regard to the aim and the background of the reporting code and the various forms and signals of DV (Table 3). However, less than half stated that they were sufficiently informed about the judicial considerations regarding their confidentiality agreement. In addition, most GDPs said that the action plan is clear with regard to the description of the different signals of DV and CA and the five steps GDPs have to take when they suspect DV or CA. However, about 40 % did not think the action plan was clear enough about this.

**Table 2:** Having taken cognizance of the RCD by GDPs (*n* = 264; 100 %)

Yes, read both the information brochure and the action plan	59 %
Yes, but only read the information brochure	11 %
Yes, but only read the action plan	6 %
No, read neither the information brochure, nor the action plan	24 %

**Table 3:** Percentage of the GDPs that regards different aspects of the information brochure and the action plan as clear^a^

Information brochure of the RCD clear about … (*n* = 185; 100 %)	
- The aim of the reporting code	84 %
- The background of the reporting code	83 %
- The various forms of DV and CA	68 %
- Signals of the various forms of DV and CA	63 %
- Judicial considerations regarding their confidentiality agreement	49 %
- The difference between asking advice and reporting a case	45 %
- The implications of reporting a case	36 %
- The function of Advice and Reporting Centers for DV and CA	35 %
Action plan of the RCD clear with regard to … (*n* = 164; 96 %)	
- The description of the signals of DV and CA	62 %
- The description of the five steps	61 %

^a^The other GDPs regard the aspect as unclear, are neutral or do not have an opinion on the matter

Table 4 shows that 51 % of GDPs have implemented the RCD in their practice in one form or another. These GDPs have, in comparison to those who did not implement, more often read the brochure and/or the action plan of the RCD (98 % versus 53 %; *p* < 0.01). They also more often answered that they are sufficiently informed about the background of the reporting code (91 % versus 70 %; *p* < 0.01), the aim of the reporting code (91 % versus 76 %; *p* < 0.05), the various forms of DV and CA (74 % versus 59 %; *p* < 0.02), the difference between asking advice and reporting a case (55 % versus 30 %; *p* < 0.01) and the implications of reporting a case (42 % versus 26 %; *p* < 0.01). Furthermore they more reported to be well informed about the five steps (67 % versus 43 %; *p* < 0.05).

**Table 4:** Implementation of the RCD in their dental practices by GDPs (*n* = 245; 93 %)

	Actions taken	Implementation
Yes, following the receiving of the RCD one or more actions were taken, namely:		51 %
- The RCD has been brought to the attention of the the practice’s staff members	40 %	
- The RCD is discussed with every staff member in the practice	25 %	
- The staff has made agreements about how to handle the RCD	13 %	
- Other form of action	3 %	
No, they have not implemented the RCD (yet), they do not use the reporting code in the practice		45 %
Differently (‘don’t know’; ‘RCD does not apply (solo practice)’; ‘not my responsibility’)		4 %

All in all, 30 % of GDPs stated that the RCD had influenced their alertness and the way in which they take action (Table 5). Almost half of the respondents (47 %) adopted a neutral stance in this regard, while 23 % answered that the influence of the RCD was limited.

**Table 5:** Influence of the RCD on their alertness and ways in which they take action according to GDPs who have implemented it (*n* = 114; 85 %)^a^

Very limited (score 3, 4)	5 %
Limited (score 5–7)	18 %
Neither limited, nor much (score 8–10)	47 %
Much (score 11–13)	27 %
Very much (score 14, 15)	3 %
Cronbach’s Alpha	0.82
Mean	9.2
Median	9.0
Mode	9.0
Standard deviation	2.6
Minimum	3.0
Maximum	15.0

Total scale range from 3 up to 15

^a^Likert scale of the opinions (from 1 ‘totaly unagree’ to 5 ‘totaly agree’) of GDPs about three (out of four) statements:

a: ‘The RCD has made me more alert with regard to signals of DV or CA’

b: ‘As a result of the RCD, I have reported suspicions of DV or CA in patients records more often’

c: ‘As a result of the RCD, I take action sooner in case of suspicions of DV or CA’

d: ‘The RCD is supportive when it comes to take action in case of suspicions of DC or CA’. This item shows minor correlation with items a, b and c and Cronbachs Alpha for all four items is 0.78. Therefore this item was not incorporated in de scale

### Suspicion of DV or CA

Twenty-four percent of the GDPs (*n* = 58) stated that they have had suspicions of DV or CA in the last 12 months in the case of 2.4 patients on average. These GDPs are more often female (52 % versus 28 %; *p* < 0.01) and on average younger (43.7 versus 51.1 years; *p* < 0.01) than GDPs who have not had any suspicions of DV or CA. Moreover, these GDPs have implemented the RCD much more often (71 % versus 45 %; *p* < 0.01).

Table 6 shows some details of the most recent case these GDPs had seen. In most cases (85 %) it involved a child or adolescent. The GDPs found signals, ranging from conspicuous signs in the mouth, lack of general care (clothing, hygiene, diet), unfriendly and humiliating behavior of the parents to signs of (inexplicable) physical harm. Most GDPs (81 %) made a note of their suspicions and/or took action in these cases (58 %). Those GDPs talked to (the parents of) the patient (52 %) in question and/or consulted a colleague or GP (33 %). Less GDPs asked for advice (24 %) or reported the case (18 %).

**Table 6:** Some information about the patient in the most recent case in which GDPs had suspicions of DV or CA (*n* = 57; 98 %)

*Patient was …*	
Child/adolescent (<18 years old)	85 %
Adult	15 %
*One or more traces or signals the GDPs noted with the patient or their parents/guardians:*	
In patient’s mouth: bad hygiene, untreated carieuze laesies, dental injury, deviance in mucosa	78 %
Appearance of patient: shabby clothing, bad hygiene, clear unhealthy diet	59 %
Behavior of parents or guardians of the patient: careless, scaring them, humiliating them, threathening, demeaning	50 %
Patient’s injury: bruises, fractures, explanation of parents or guardians that does not fit the injury, deviant behavior	50 %
*Actions taken by the dentist:*	
Yes, have made a note in the patient’s record and have taken other actions	49 %
Yes, have only made a note in the patient’s record	32 %
Yes, have only taken other actions	9 %
No, have neither made a note in the patient’s record, nor taken other actions	10 %

GDPs who did not take action said they felt unsure about whether or not their suspicions were correct (88 %). GDPs who wrote down their suspicions of DV or CA in the patient’s record and/or took action are, in comparison to those who did not, more often aware of the RCD (86 % versus 43 %; *p* < 0.01) and have implemented it in their practice more often (84 % versus 0 %; *p* < 0.01).

### E-learning module

Sixty-one percent of GDPs wants to complete the e-learning module ‘The Next Page’ and/or thinks it is important that their team complete the module (Table 7). These GDPs are, compared to other GDPs, more often female (39 % versus 24 %; *p* < 0.05). Moreover, they have implemented the RCD more often (61 % versus 36 %; *p* < 0.01) and they have had suspicions of DV or CA in the last 12 months (32 % versus 11 %; *p* < 0.01). In more cases, they take a positive stance with regard to the legal obligation to use the reporting code (66 % versus 35 %; *p* < 0.01). The RCD also has had more influence on them (9.8 versus 7.1; *p* < 0.01).

**Table 7:** Plan of GDPs (*n* = 238; 90 %) to complete the e-learning method ‘The next page’ and the wish for their team (oral hygienist, dental assistant) to complete the method as well

GDP wants to complete the method and thinks it is important for the team to do the same	46 %
GDP wants to complete the method or thinks it is important for the team to complete the method	15 %
GDP is unsure about whether or not to complete the method or the importance of the team to complete the method	29 %
GDP does not want to complete the method and does not think it is important for the team to complete the method	10 %

## Conclusions and discussion

This study shows that a large majority of GDPs was aware of the obligation to use a reporting code in 2014. Over 50 % of GDPs viewed this as positive. Those who were not positive usually felt unsure about the issue of DV and dealing with it. Most GDPs are also aware of the RCD. The majority of GDPs who have had suspicions of DV or CA took some form of action.

The study gives some insight in why almost half of the GDPs have not implemented the reporting code into their practice. It appeared that these GDPs more often failed to take notice of the RCD than those who implemented the code. Moreover, there seems to be a connection between failure to implement and the information in the RCD. After all (proportionately) many GDPs who did not implement stated that the RCD insufficiently informed them about the background and aim of the reporting code, about the various forms of DV and CA, about aspects regarding asking advice and reporting a case and about the five steps they have to take.

The information in the RCD is apparently not able to clarify the issue to all GDPs. Admittedly, Dutch legislation is complicated. GDPs, even though they are not qualified to diagnose DV or CA, are expected to judge suspicions based on their own observations and to follow the guideline which consists of five rather difficult steps. This starts with ‘identifying the signs’. According to this study, a large number of GDPs are unsure about the correctness of their suspicions. They are required to talk about their suspicions with experts. But the apparent unclarity regarding the task and the function of the Advice and Reporting Centers can form a barrier here and the same holds true for the judicial aspects of the confidentiality agreement. The next step is talking to the patient or their parents. This is not an easy task either when it comes to suspicions of DV or CA. Lastly, the GDP is supposed to decide whether or not to report his or her suspicions. What is best for the patient? How can I make sure not to damage the relationship with the patient and between the patient and their family? And are the signals strong enough to justify taking action? As becomes clear from the literature, GDPs and caretakers from other countries are also wrestling with these problems. For example Scottish GDPs, whose failure to take action is related to lack of certainty of the diagnosis [19] and or fear for family violence towards the child or towards themselves [10]. Another example is the fact that primary healthcare clinicians in the United Kingdom, part of whom are insufficiently prepared to ask patients appropriate questions about DV or to identify signs and symptoms of DV [17]. In other words, the problems of the Dutch GDPs are relatable.

This does not change the fact that the reporting code is a legal obligation and that the inspectorate can ask individual GDPs to provide concrete details of how they implemented the code in their practice, what they have done to train the dental team in order to promote awareness and use of the reporting code and what their plans are for the coming year. It is therefore important for GDPs to be aware of their obligations. The KNMT should inform GDPs about these matters. Offering practical support with regard to using the RCD is also of pivotal importance when it comes to helping the GDP and the dental team in the diagnosis and documentation [12]. After all, this study shows that a reporting code can have the intended effect. It appears that GDPs who properly implemented the RCD have discerned signs of DV and CA more often and they have also acted on these suspicions.

Furthermore, it appears that educational interventions such as online tutorials and an e-learning module seem to have positive effects in particular when it comes to knowledge of DV and recognizing it [30–32]. Based on literature it can be assumed that the e-learning method the KNMT offers is a good initiative [33, 34]. Maybe this method can also create a better understanding of the different steps in the reporting code.

Moreover, the Mandatory Reporting Code DV and CA makes it compulsory for health professionals like GDPs to promote the awareness and use of the code within their practice. This means that by offering an e-learning method the KNMT meets these educational needs. Most GDPs appear to appreciate this initiative.

Finally, a critical evaluation of the results of this study is needed. The relatively high number of non-response raises questions. Regardless of a certain ‘research fatigue’ GDPs may experience (which plays a role in every survey), the subject of this research may also have been a reason not to respond. For example because of disinterest, but more importantly, insecurities regarding how to handle suspicions of DV and CA and fear of acting legally insufficient may play a role. Presumably, the percentage of GDPs in the Netherlands that have not implemented the reporting code in their practice is higher than the 49 % this study found. In relation to this, a certain amount of ‘socially acceptable answers’ should be taken into consideration. This is likely with regard to for example implementing the reporting code in their practice as well as taking action when GDPs have suspicions of DV or CA.

All in all, it can be concluded that the large number of GDPs who do not (yet) comply with the new legislation do not fail to do so because of unwillingness, but merely because of inexperience and insecurities with regard to handling signs of DV and CA. This survey shows that a reporting code like the RCD can be a good support to GDPs and can encourage them to be alert on signs of DV and to report these. Moreover, the survey shows that the KNMT should pay close attention to the ways in which GDPs are coping with their legal obligations and to possible problems that may arise. Meanwhile, the legislation in the Netherlands has been adjusted in a number of ways. The KNMT is working on an updated version of the RCD, which will be published in 2015. In this version, partly in response to the conclusions of this study, clear information on the new legislation and steps that need to be taken is of pivotal importance.

In the future the KNMT should investigate whether or not GDPs are better informed as a consequence of the updated RCD and whether or not they meet the legal requirements. In this context, the KNMT should also take stock of possible problems regarding dealing with (suspicions of) DV and CA. However, this is also a public health issue, and more attention should be paid to making this an issue of public interest. When society regards these issues as highly important, it will be easier for GDPs to implement this new legislation in their daily practice. Future research should then focus on the effect of (early) reporting DV and CA. A complicating factor is that on January 1^st^ 2015 the Dutch government transferred the responsibility for the approach of DV and CA to municipalities. They have to make their own agreements with certified agencies responsible for child protection measures in their region, which leads to regional differences. Fortunately, the Dutch government is aware of the importance of monitoring this issue. In 2015, the Ministry of Health Welfare and Sport (VWS) ran a quick scan in order to investigate the workings of the Mandatory Reporting Code DV and CA. This quick scan should provide insight into how professionals experience work with the Reporting Code.

## Abbreviations

CA: child abuse
DV: domestic violence
GDPs: general dental practitioners
IGZ: Dutch Health Care Inspectorate
KNMT: Royal Dutch Dental Association
RCD: reporting code for dental professionals
